# Factors associated with antithrombotic treatment decisions for stroke prevention in atrial fibrillation in the Stockholm region after the introduction of NOACs

**DOI:** 10.1007/s00228-017-2289-0

**Published:** 2017-06-29

**Authors:** Joris Komen, Tomas Forslund, Paul Hjemdahl, Björn Wettermark

**Affiliations:** 1 Centre for Pharmacoepidemiology, Department of Medicine Solna, Karolinska Institute, Karolinska University Hospital, T2, 171 76 Stockholm, Sweden; 2 Department of Pharmaceutical Sciences, Utrecht University, Universiteitsweg 99, 3584 CG Utrecht, the Netherlands; 3 Public Healthcare Services Committee, Department of Healthcare Development, Stockholm County Council, Box 6909, 102 39 Stockholm, Sweden; 4 Department of Medicine Solna, Clinical Epidemiology/Clinical Pharmacology, Karolinska Institutet and Karolinska University Hospital, L7:03, 171 76 Stockholm, Sweden

**Keywords:** Atrial fibrillation, Antithrombotic treatment, NOACs, Stockholm

## Abstract

**Purpose:**

The purpose of this study was to investigate the influence of patient characteristics such as age and stroke and bleeding risks on decisions for antithrombotic treatment in patients with atrial fibrillation (AF).

**Methods:**

This was a retrospective, population-based study including AF patients initiated with either warfarin, dabigatran, rivaroxaban, apixaban, or low-dose aspirin (ASA) between March 2015 and February 2016. Multivariate models were used to calculate adjusted odds ratios (aOR) for factors associated with treatment decisions.

**Results:**

A total of 6765 newly initiated patients were included, most with apixaban (46.4%) and least with ASA (6.7%). There were more comorbidities in patients initiated with ASA or warfarin compared to the cohort average. Patients with high stroke risks had higher chances of receiving ASA (CHA_2_DS_2_-VASc ≥5 vs 0; aOR 2.01; 95% confidence interval (CI) 1.12–3.33). Among patients receiving oral anticoagulants, patients with high bleeding risks more often received warfarin (ATRIA score 5–10 vs 0–3; aOR 1.40; CI 1.20–1.64). Among NOACs, apixaban was preferred for patients with higher stroke risks (aOR 1.78; CI 1.31–2.41), high bleeding risks (aOR 1.54; CI 1.26–1.88) and high age (age group ≥85 vs 0–65; aOR 1.84; CI 1.44–2.35). Conversely, dabigatran treatment was associated with lower ages and lower risks.

**Conclusions:**

High stroke and bleeding risks favored choices of warfarin or ASA. Among patients receiving NOACs, apixaban was favored for elderly and high-risk patients whereas dabigatran was used in lower risk patients. The inadvertent use of ASA, especially among those with high stroke risks, should be further discouraged.

**Electronic supplementary material:**

The online version of this article (doi:10.1007/s00228-017-2289-0) contains supplementary material, which is available to authorized users.

## Introduction

Patients with atrial fibrillation (AF) on average have a fivefold increased risk for stroke compared to the general population [1]. Treatment with oral anticoagulants reduces this risk by two thirds [2]. With a prevalence of more than 3% in the total adult population in Sweden, AF is the most common arrhythmia, with more than 80% of the patients having risk factors motivating chronic oral anticoagulant therapy [3].

In 2011, the first of the presently available non-vitamin K oral anticoagulants (NOACs), dabigatran, was registered in Europe for the prevention of thromboembolic complications in patients with AF [4]. Rivaroxaban and apixaban [5, 6]were registered for thromboembolic prophylaxis in patients with AF and reimbursed on the Swedish market in 2012 and 2013, respectively. NOACs are effective alternatives to the traditional treatment with vitamin K antagonists like warfarin and are now extensively used [7].

The efficacy and safety of these NOACs compared to warfarin have been demonstrated in one pivotal phase III clinical trial for each drug [8–10], but the effectiveness and safety of drugs may differ substantially between clinical trials and clinical practice [11]. The risk-benefit ratio of treatment with a NOAC or warfarin may, e.g., depend on the population treated, with important discrepancies between the trial populations and real-life users of these drugs [12].

Low-dose aspirin (ASA) has been shown to be much less effective than oral anticoagulant therapy for stroke prevention in AF without being safer from the standpoint of bleeding [2, 13, 14], and several guidelines recommend ASA only for AF patients who are unwilling or unable to take oral anticoagulation treatment [15, 16]. Nonetheless, ASA is still used by a substantial number of AF patients [17] for reasons which are not fully understood but may reflect physicians’ reluctance to change therapeutic traditions or misperceptions regarding the benefit and safety of ASA treatment.

Previous studies have shown important factors associated with the prescribing of either warfarin or a NOAC [12, 18–21], but there is limited knowledge regarding predictors for prescribing ASA or for decisions between the three NOACs. The aim of the current study was to investigate the influence of patient characteristics such as stroke risk, bleeding risk, and age on decisions regarding antithrombotic treatment in patients with atrial fibrillation.

## Methods

### Patient selection

For this retrospective, population-based study, we used the administrative health register of the Stockholm County (Vårdanalysdatabasen, the VAL database). Pseudonymized data regarding patient sex, age, diagnoses, prescription claims, hospitalizations, and other healthcare consultations, migration and death for all 2.2 million inhabitants in the Stockholm region are available in the database [22], and may be linked through the Personal Identity Number [23]. All diagnosis codes from primary care, hospitalizations, and specialist consultations in ambulatory care are included. Since July 2010, the VAL database also includes data on claims of prescriptions from any pharmacy in Sweden corresponding to the information available in the National Swedish Prescribed Drug register, i.e., amounts, expenditures and reimbursement, the age and sex of the patient, co-payments and prescriber category [24].

The analyses were conducted in VAL, which is the administrative health care register of the Stockholm region [22]. The data in VAL is pseudonymized and individual patients cannot be identified. The research was approved by the Regional Ethical Review Board in Stockholm and personal data permit was obtained from the Public Healthcare Services Committee, Department of Healthcare Development of the Stockholm County Council.

We included all first claimed prescriptions from March 2015 to February 2016, of either warfarin (ATC: B01AA03), low-dose ASA (ATC: B01AC06), or a NOAC (ATC: B01AE07, B01AF01 or B01AF02) after a 9-month wash out period to identify newly initiated patients. Patients were excluded if there was no registered diagnosis code for AF (see Appendix Table 1 for ICD codes) from 2003 until the date of the first claim of the antithrombotic agent selected for the patient. Initiations were also excluded if the patient had a recorded procedure code for mechanical valves, or a diagnosis code for mitral stenosis. Patients were then excluded if they had been treated with any oral anticoagulant 6 months prior to initiation. Comorbidities and prescriber information were linked to the initiations using ICD-10 codes recorded at each consultation and prescriber codes recorded at the first prescription, respectively [23].

**Fig. 1:**
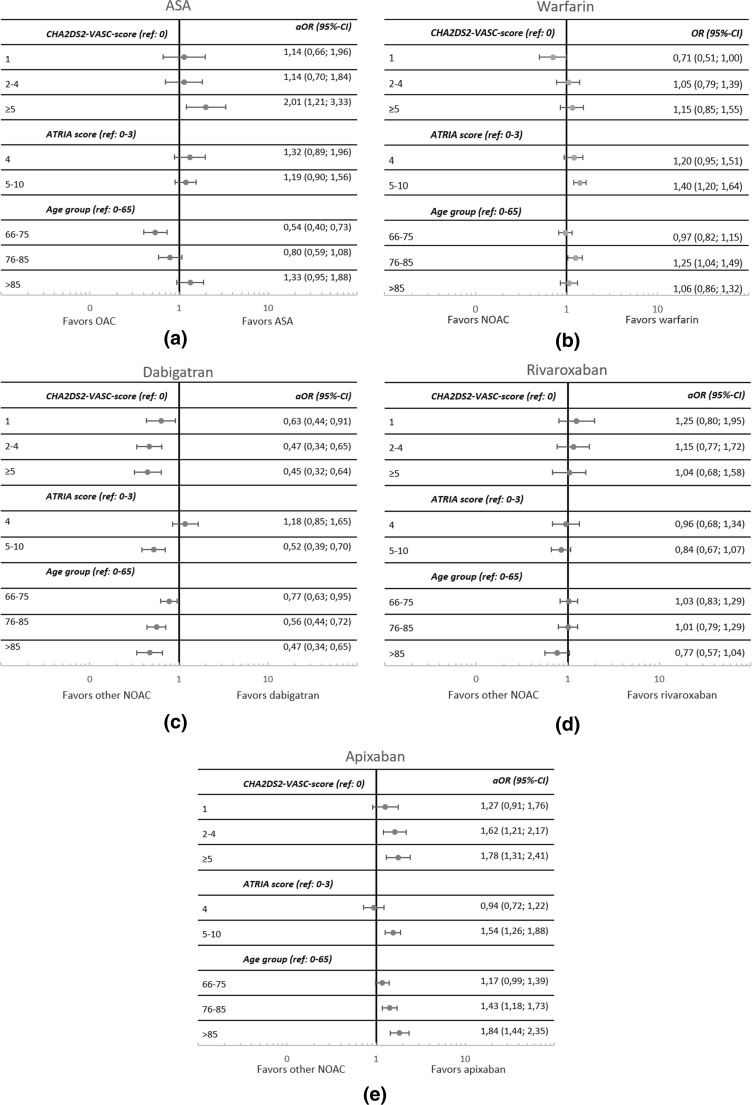
Adjusted odds ratios (aOR) of factors associated with treatment decisions for **a** ASA compared to an oral anticoagulant, **b** warfarin compared to a NOAC, **c** dabigatran compared to apixaban and rivaroxaban, **d** rivaroxaban compared to dabigatran and apixaban, and **e** apixaban compared to dabigatran and rivaroxaban. Three multivariate models were used to calculate how stroke risk, bleeding risk, and age group influenced treatment decisions

From this cohort, we created three subgroups of patients initiated on the drugs included in the analyses (Fig. 1). The first subgroup was created to analyze predictors for treatment with ASA versus any oral anticoagulation treatment, the second subgroup was created for analyses comparing warfarin and NOAC, and the third subgroup to analyze predictors for decisions of the three NOACs separately. In the first subgroup, we excluded patients who had been treated with ASA 6 months prior to inclusion to avoid including patients twice in the same analyses. In the second and third subgroups, patients could be treated with ASA prior to inclusion.

For the patient’s comorbidities, we searched for registered diagnostic codes by any caregiver in the region from 2003 until the date of inclusion. Ischemic stroke risks were evaluated by calculating the CHA_2_DS_2_-VASc scores [25] (congestive heart failure +1, hypertension +1, age [65–74 + 1; ≥75 years +2], diabetes mellitus +1, previous ischemic stroke +2, vascular disease +1, and female sex +1). Bleeding risks were calculated using the ATRIA score (anemia +3, severe renal disease +3, age ≥ 75 + 2, any prior hemorrhage diagnosis +1 and hypertension +1) [26]. The age of each patient was determined at the date of inclusion. Other comorbidities included in the models, defined in Table 1 as complicating comorbidities, were chosen based on previous knowledge and standards from published studies.

**Table 1:** Baseline characteristics of patients newly initiated with treatment from March 2015 until February 2016. All numbers are percentages unless otherwise stated

Variable	Overall	ASA	Warfarin	Dabigatran	Rivaroxaban	Apixaban	*p* value
Number of patients	6765	453	1691	717	770	3134	
Male sex	54.7	54.1	54.9	60.4	54.4	53.4	0.022
Age							
Mean age (years)	74.3	75.1	74.9	70.4	73.7	74.8	<0.001
0–65	20.3	24.9	17.9	29.7	20.9	18.6	
66–75	32.2	20.1	30.5	37.7	35.2	32.8	
76–85	30.1	28.3	33.9	22.7	29.6	30.1	
≥86	17.5	26.7	17.6	9.9	14.3	18.5	
CHA_2_DS_2_-VASc score							
CHADSVASc (mean)	3.67	3.77	3.89	3.17	3.56	3.69	<0.001
0	4.4	4.4	4.1	8.6	4.0	3.7	<0.001
1	9.9	10.6	7.2	14.4	11.6	9.9	
2–4	52.5	47.9	52.5	51.2	53.9	53.2	
≥5	33.2	37.1	36.3	25.8	30.5	33.2	
Comorbidities included in CHA_2_DS_2_-VASc score							
Chronic heart failure	26.4	30.5	30.7	17.0	25.8	25.8	<0.001
Hypertension	70.2	65.6	73.9	63.7	69.1	29.4	<0.001
Age ≥ 75	50.6	56.5	54.9	34.5	47.7	51.9	<0.001
Age 65–74	31.8	20.5	29.2	38.9	34.8	32.5	<0.001
Diabetes mellitus	19.2	17.0	23.1	15.3	19.1	18.3	<0.001
Stroke or embolism	22.4	24.7	20.8	26.2	19.5	22.8	0.007
Vascular disease	28.3	35.5	35.2	21.2	27.5	25.3	<0.001
Female sex	45.3	45.9	45.1	39.6	45.5	46.6	0.022
ATRIA score							
ATRIA (mean)	2.6	2.9	2.9	1.9	2.4	2.6	<0.001
0–3	76.7	70.4	72.0	85.5	79.9	77.3	<0.001
4	6.2	7.9	6.8	6.7	6.0	5.6	
≥5	17.1	21.6	21.2	7.8	14.2	17.1	
Comorbidities included in ATRIA score							
Anemia	17.3	21.4	20.4	11.3	15.7	16.9	<0.001
Renal disease	8.7	11.7	13.2	3.2	6.4	7.7	<0.001
Age ≥75	50.6	56.5	54.9	34.5	47.7	51.9	<0.001
Serious bleeding	9.8	12.1	9.7	8.9	7.8	10.2	0.106
Hypertension	70.2	65.6	73.9	63.7	69.1	29.4	<0.001
Prescriber category							
Primary care	31.8	28.0	48.8	18.7	30.9	26.5	<0.001
Cardiology	26.5	14.3	17.8	33.2	29.4	30.7	<0.001
Internal medicines	19.4	14.6	13.8	19.2	19.1	23.3	<0.001
Geriatrics	7.6	15.5	8.2	5.2	4.5	7.5	<0.001
Other/unknown	14.7	27.6	11.4	23.7	16.1	12.0	<0.001
Complicating comorbidities							
Liver disease	2.1	4.6	2.5	1.3	2.9	1.6	<0.001
Dementia	5.0	11.3	2.8	2.5	6.1	5.5	<0.001
VTE	9.6	5.7	10.6	6.8	15.5	8.8	<0.001
Alcoholism	6.3	8.6	5.8	7.5	6.5	5.8	0.097
Cancer	24.8	26.7	25.6	19.4	23.2	25.6	0.005
COPD	10.7	11.3	10.9	7.8	9.7	11.4	0.069
Frequent falls	15.7	19.2	15.1	12.1	17.4	15.9	0.010
Obesity	9.8	7.9	10.4	11.2	10.3	9.3	0.290

### Statistical analyses

Descriptive statistics were used to describe the baseline characteristics of the treatment groups. One-way analyses of variance (ANOVA) were used to calculate *p* values for differences between mean values. For variables with proportional values, a chi-square test was used. We analyzed factors associated with ASA treatment compared to oral anticoagulant treatment, warfarin compared to NOAC, and one NOAC compared to the two other NOACs. In a multivariate model, we calculated adjusted odds ratios (aOR) with 95% confidence intervals (CI) for treatment decisions for different stroke risk, bleeding risk, and age categories. Variables in the multivariate model were chosen based on previous knowledge and standards from published literature. To investigate the effects of the CHA_2_DS_2_-VASc score and the ATRIA score on treatment decisions, we adjusted for gender and all comorbidities presented in Table 1, except for the qualifying risk factors of the scores. For the effect of the age group on the treatment decision, we adjusted for gender and all comorbidities presented in Table 1, and with this model, we could therefore investigate each qualifying comorbidity from the stroke and bleeding risk calculation. We checked all models for statistically significant interactions between the covariates. The statistical package IBM SPSS Statistics version 23.0 was used for all statistical analyses. Data extraction was performed using SAS EG 6.1 (SAS Institute Inc., Cary, NC).

## Results

### Patient selection

A total of 6765 patients were included in the cohort (see Appendix Figure 1). The first subgroup comparing ASA with oral anticoagulant therapy consisted of 4316 patients previously not treated with ASA, the second subgroup comparing warfarin versus NOACs of 6312 patients, and the third subgroup comparing the three NOACs consisted of 4621 patients.

### Baseline characteristics

Among the patients initiated with oral anticoagulant treatment, 27.8% received warfarin and 72.2% received a NOAC (Table 1). Among patients treated with a NOAC, 15.5% received dabigatran, 16.7% rivaroxaban, and 67.8% apixaban. The mean age of the cohort was 74.3 years and 54.7% were males. The proportions of patients with the highest risks for stroke and bleeding were higher in patients initiated with ASA or warfarin; the group initiated with ASA had the highest proportion of very old patients as well (Table 1). The mean CHA_2_DS_2_-VASc and ATRIA scores were the lowest for patients initiated with dabigatran, while patients initiated with apixaban and rivaroxaban differed little from the cohort average. The proportions of patients with renal disease and anemia were the lowest among dabigatran-initiated patients. The baseline characteristics of patients in the different treatment groups did not differ between 2014 (Appendix Table 2), i.e., before regional recommendations regarding NOACs were issued, and the study period (Table 1).

Warfarin was preferentially prescribed in primary care, while ASA was prescribed more often by geriatricians and dabigatran by cardiologists. Regarding comorbidities, dementia was less common in patients initiated with warfarin or dabigatran, VTE was more common in patients with rivaroxaban, and all comorbidities, except VTE and obesity, were more common than average in patients initiated with ASA.

The prevalence of vascular disease (i.e., angina pectoris, myocardial infarction, atherosclerosis, and peripheral vascular disease) was higher among patients treated with ASA compared to oral anticoagulation in the elderly (34.7 vs. 28.0%) and among patients with a CHA_2_DS_2_-VASc score ≥5 (51.8 vs. 41.8%).

Of the patients initiated with warfarin, 45.9% had been treated with ASA in the 6 months prior to inclusion, 8.9% with clopidogrel and 6.5% with both ASA and clopidogrel. For patients initiated with a NOAC, these were 36.2, 4.0, and 1.5%, respectively.

### Factors associated with treatment

#### ASA and warfarin

A high stroke risk increased the probability of receiving ASA instead of oral anticoagulant treatment, while stroke risk did not influence the probability of receiving warfarin compared to a NOAC (Figs. 1a, b). A high bleeding risk drove the decision from a NOAC towards warfarin while the bleeding risk did not influence the decision for ASA. Age did not play a substantial role in the decision between warfarin or a NOAC. Patients in the age group 66–75 years had a decreased probability of receiving ASA compared to an oral anticoagulant, but this probability was increased among the very old.

Comorbidities associated with an increased use of ASA were liver disease, dementia, and vascular disease, while VTE drove the decision towards oral anticoagulant treatment (Appendix Figure 2A). Renal disease and vascular disease favored warfarin, and dementia favored NOAC treatment (Appendix Figure 2B).

#### NOACs

Among patients treated with a NOAC, the chances of being treated with apixaban were higher for patients with higher risks for stroke and bleeding and in higher age groups (Fig. 1e), while the chances of receiving dabigatran were lower for patients in these groups (Fig. 1c). Initiations of rivaroxaban were not specifically associated with either stroke risk, bleeding risk, or age group (Fig. 1d). In the age category models, the probability of receiving dabigatran was increased if patients had a previous stroke or thromboembolism, while renal disease and dementia decreased the probability (Appendix Figure 2C). Liver disease and VTE increased the probability of receiving rivaroxaban and renal disease favored apixaban (Appendix Figures 2D and 2E).

## Discussion

In this retrospective population-based study, we found that stroke risk, bleeding risk, and age category influenced the prescribers’ treatment decision for stroke prevention in AF patients in a manner which was not always in accordance with the evidence base and recommendations. Patients with the highest stroke risk had an increased probability of receiving ASA treatment, while bleeding risk did not influence this decision. The probability of receiving ASA was decreased in patients aged 66–75 but increased in the very old. For warfarin, the decision was driven by higher bleeding risks, while stroke risk and age did not influence the probability of prescribing warfarin. Among patients initiated on a NOAC, higher stroke risk, higher bleeding risk, and higher age drove decisions towards apixaban and away from dabigatran, while the probability of receiving rivaroxaban was not influenced by these variables. The pattern of choices between NOACs did not seem to be influenced by the introduction of regional NOAC recommendations in 2015 but there was a large increase in apixaban prescribing after 2014.

Especially ASA, and to a lesser extent warfarin, continued to be chosen for more severely ill patients after the introduction of the NOACs. Thus, almost all comorbidities were more common among ASA- and warfarin-initiated patients compared to the cohort average. This indicates that prescribers tend to stay with well-known old drugs for the treatment of more vulnerable patients. Despite clear-cut recommendations since several years to favor oral anticoagulant over ASA treatment, 6.7% of all patients were still initiated with ASA. It is especially remarkable that patients with the highest risks for stroke, to a large part driven by high age, more often received ASA compared to patients with lower risks, since ASA is much less effective than oral anticoagulation treatment for the prevention of stroke without offering significant benefits regarding safety [2, 13, 14]. However, the higher prevalence of vascular disease among the elderly and high-risk patients could have contributed to this decision. We have no data on stent placements which to some extent could contribute to ASA treatment. Patients could potentially be treated with single or dual antiplatelet therapy plus oral anticoagulation at the time of inclusion. However, there is a difficulty in correctly identifying patients who switched and those actually receiving this combination therapy. Still, it seems as if the combination occurred more often in patients treated with warfarin, indicating again that this was the preferred therapy for the more severely ill patients.

Among geriatricians, the proportions initiated on ASA or warfarin were larger than the cohort average. This could in part be due to uncertainty about still poorly investigated drug-drug interactions with NOACs among elderly frail patients with many drugs [27]. Warfarin was the preferred alternative for initiation of oral anticoagulation in primary care indicating that the uptake of NOACs, as for other new drugs, is dependent on acceptance in secondary care before becoming established in primary care [28].

The ROCKET-AF trial included only patients with a CHADS_2_ score of 2 or above (equivalent to CHA_2_DS_2_-VASc scores well above 3), which resulted in a mean CHADS_2_ score of 3.5 and the oldest patient population among the pivotal NOAC trials [9]. However, in our real-life users, the average CHADS_2_ score for rivaroxaban-treated patients was only 2.1 (data not shown). Instead, apixaban was the favored NOAC for older patients with higher risks for stroke. This suggests that factors other than trial characteristics guide the prescribers in their choice of NOAC for high-risk patients. Local recommendations in the Wise List prioritized apixaban among the NOACs during the study period [7], but the patient characteristics in the different treatment groups were similar the year before this recommendation (Appendix Table 2). Previous studies report similar results with rivaroxaban being initiated in patients with average CHADS_2_ scores of 2.7 [21] and 1.7 [29]*.* Due to the lack of randomized clinical trial data for rivaroxaban in patients with CHADS_2_ scores 0–1, further investigation of the risk-benefit ratio for this drug in low-risk AF patients is of interest. Similarly, since the ARISTOTLE trial included a substantially smaller proportion of elderly patients than that found among real-life users, close follow-up of elderly high-risk patients is needed [10].

To our knowledge, this is the first study of patient characteristics associated with decisions between the available NOACs as well as decisions to resort to ASA instead of an oral anticoagulant. Some studies have determined predictors for NOAC compared to warfarin treatment [12, 18–21] and found that higher stroke and bleeding risks often are associated with warfarin use. In the present, more recent study, a higher stroke risk did not channel the selection towards warfarin, indicating that the experience gained by prescribers has enabled the use of NOACs also in higher-risk patients. This is in accordance with guidelines either favoring NOACs over warfarin (ESC) or giving them equal priority (US and Swedish). However, warfarin treatment was still favored for patients with higher bleeding risks, most likely due to the possibility to personalize warfarin treatment and the availability of well-established drugs and routines for reversal of bleeds related to warfarin treatment, whereas specific NOAC antidotes were still lacking.

Dabigatran was preferentially used among younger, low-risk patients. The dependence on renal function for the elimination of dabigatran [4] has apparently been an important factor when choosing an oral anticoagulant for elderly AF patients. Dabigatran is the recommended second-line NOAC in the regional recommendations in Stockholm but should be used with caution for elderly and frail patients who often have renal impairment [15]. Dabigatran drug levels can be measured in routine care in Stockholm but the possibility to monitor if the dosage is adequate is seldom used; it appears to be simpler for the prescribers to choose another drug which is thought to be safer for vulnerable patients than to individualize the dose. Apixaban is the NOAC which is least dependent on renal function for its elimination [4–6]. This might explain why elderly patients who often have renal impairment were more likely to receive apixaban and less likely to receive dabigatran.

Our study has some limitations. Firstly, previous studies have found predictors for treatment decisions for which we have no data, for example the ethnicity of the patient and the preference of the patient and/or the prescriber [12, 18, 21]. Other studies have also found regional differences in the odds for receiving NOACs or warfarin, whereas our study was confined to one region; cross-regional and cross-national comparisons would be of interest as local recommendations and routines may differ. Of interest is that patterns in NOAC prescribing changed after the introduction of regional NOAC recommendations in Stockholm in 2015 [7, 15] whereas the patient characteristics in the different treatment groups did not change after the recommendation. A limitation in the broad application of the present findings may have been created by the regional recommendations. Our previous work has shown that the regional recommendations increased apixaban prescribing and thus, to some extent choices, between NOACs but not other treatment decisions [7]. However, the pattern of patient characteristics did not change from the period before the recommendations were issued and there is no limitation on the broad application of the findings for ASA versus OAC treatment or warfarin versus NOAC treatment. Secondly, when calculating the ATRIA score, we probably underestimated renal impairment, since only limited data on renal impairment (no creatinine levels, only diagnostic codes) are available in the VAL database [30]. However, the same underestimation occurred for all patients and treatment alternatives; we believe that this possible bias is limited and that the available data allow us to interpret how bleeding risks influence prescriber decisions. Lastly, since patients were included in this cohort after being newly initiated with a treatment, we lack patients who received no treatment at all, which in some cases might be the appropriate action.

The strengths of the present study compared to previous ones are that it has been undertaken in all patients with AF in an entire healthcare system, including both primary and secondary care. This is the first study which compares predictors for all treatment alternatives, and for the three NOACs separately. Large changes have occurred in NOAC utilization in the last few years. We only investigated patients initiated from March 2015 to February 2016, since the utilization patterns and factors influencing them were relatively stable during this period [7].

In conclusion, we found that high stroke and bleeding risks favored treatment with warfarin or ASA, the latter being at odds with the available evidence and recommendations. Among NOACs, apixaban use was channeled towards high-risk patients, while dabigatran was mainly prescribed for low-risk patients. Even though rivaroxaban was tested in and marketed for high-risk patients, this did not influence the prescriber’s decision between the NOACs. Thus, post-marketing surveillance is needed to follow how patient characteristics influence prescriber’s decisions and the outcomes achieved with the treatments chosen. Increased efforts to reduce ASA treatment instead of oral anticoagulant treatment are warranted, as well as improved education and further evidence regarding the treatment of high-risk AF patients.

## Electronic supplementary material

Appendix Table 1 (DOCX 13 kb)

Appendix Table 2 (DOCX 13 kb)

Appendix Figure 1 Flow chart of patient selection (GIF 1 kb)

High Resolution Image (TIFF 78 kb)

Appendix Figure 2 Adjusted Odds Ratios (aOR) of all comorbidities associated with treatment choises for (A) ASA compared to an oral anticoagulant, (B) warfarin compared to NOAC, (C) dabigatran compared to apixaban and rivaroxaban, (D) rivaroxaban compared to dabigatran and apixaban, and (E) apixaban compared to dabigatran and rivaroxaban. In this Figure the multivariate model for analyzing the effects of age on treatment decisions is shown. This model includes all complicating comorbidities as defined in Table 1. (GIF 941 bytes) (GIF 943 bytes) (GIF 932 bytes) (GIF 948 bytes) (GIF 941 bytes)

High Resolution Image (TIFF 34 kb) High Resolution Image (TIFF 35 kb) High Resolution Image (TIFF 35 kb) High Resolution Image (TIFF 34 kb) High Resolution Image (TIFF 34 kb)
