# Supplementary material for: Factors associated with antithrombotic treatment decisions for stroke prevention in atrial fibrillation in the Stockholm region after the introduction of NOACs
Source: Eur J Clin Pharmacol. 2017 Jun 29;73(10):1315–22. doi: 10.1007/s00228-017-2289-0 (PMC5612279; doi:10.1007/s00228-017-2289-0)
Supplement: Supplementary file 1 — (DOCX 13 kb) [file 228_2017_2289_MOESM1_ESM.docx]

Appendix table 1. ICD-10 codes

| **Diagnosis** | **ICD-code beginning with** |
| --- | --- |
| **Anemia** | **D50-64** |
| **Atrial fibrillation** | **I48** |
| **Cancer** | **entire C-series** |
| **Chronic heart failure** | **I50** |
| **COPD** | **J43-44** |
| **Dementia** | **F00-F03** |
| **Diabetes mellitus** | **E10-E14** |
| **Frequent falls (more than one registration)** | **W00-19** |
| **Hypertension** | **I10-I15** |
| **Liver disease** | **K70-77** |
| **Mechanical valve** | **Procedure codes FCA60, FCA70, FDC10, FGE00, FGE10, FGE20, FGE96, FJF00, FJF10, FJF12, FJF20, FJF96, FKD00, FKD10, FKD20, FKD96, FMD00, FMD10, FMD12, FMD13, FMD20, FMD30, FMD40, FMD96** |
| **Mitral stenosis** | **I050, I052, I342** |
| **Obesity** | **E65-66** |
| **Renal disease** | **N17-19** |
| **Serious bleeding** | **I60-62, I690-I692, S064-S066, I850, I983, K25-28 (subcodes 0-2 and 4-6 only), K625, K922, D629** |
| **Stroke or embolism** | **I63, I64, I679, I693, I694, I698, I67-, I69-, Z866, Z867, G450, G451, G452, G453, G458, G45.9, G45-, I74** |
| **Vascular disease** | **I20-I25, I70, I739** |
| **Venous thromboembolism** | **I26, I80-I82** |

Appendix table 2. ICD-10 codes used for defining comorbidities in the database
