# Supplementary material for: Factors associated with antithrombotic treatment decisions for stroke prevention in atrial fibrillation in the Stockholm region after the introduction of NOACs
Source: Eur J Clin Pharmacol. 2017 Jun 29;73(10):1315–22. doi: 10.1007/s00228-017-2289-0 (PMC5612279; doi:10.1007/s00228-017-2289-0)
Supplement: Supplementary file 2 — (DOCX 13 kb) [file 228_2017_2289_MOESM2_ESM.docx]

Appendix Table 2

| **Variable** | **Overall** | **ASA** | **Warfarin** | **Dabigatran** | **Rivaroxaban** | **Apixaban** |
| --- | --- | --- | --- | --- | --- | --- |
| **Patients initiated in 2014** | | | | | | |
| ***Number of patients*** | 6 576 | 713 | 3 099 | 1 036 | 924 | 795 |
| ***Male sex*** | 55.6 | 53.9 | 54.4 | 61.3 | 53.9 | 56.2 |
| ***Age*** | | | | | | |
| ***Mean age (years)*** | 74.2 | 75.5 | 74.8 | 69.8 | 74.6 | 75.5 |
| ***CHA_2_DS_2_-VASc-score*** | | | | | | |
| ***CHADSVASc (mean)*** | 3.64 | 3.64 | 3.79 | 2.96 | 3.73 | 3.82 |
| ***ATRIA-score*** | | | | | | |
| ***ATRIA (mean)*** | 2.5 | 2.8 | 2.7 | 1.8 | 2.4 | 2.7 |
| **Population in the cohort** | | | | | | |
| ***Number of patients*** | 6 765 | 453 | 1 691 | 717 | 770 | 3 134 |
| ***Male sex*** | 54.7 | 54.1 | 54.9 | 60.4 | 54.4 | 53.4 |
| ***Age*** | | | | | | |
| ***Mean age (years)*** | 74.3 | 75.1 | 74.9 | 70.4 | 73.7 | 74.8 |
| ***CHA_2_DS_2_-VASc-score*** | | | | | | |
| ***CHADSVASc (mean)*** | 3.67 | 3.77 | 3.89 | 3.17 | 3.56 | 3.69 |
| ***ATRIA-score*** | | | | | | |
| ***ATRIA (mean)*** | 2.6 | 2.9 | 2.9 | 1.9 | 2.4 | 2.6 |

Table 1 Comparing patient characteristics of the population in the cohort to those initiated in 2014, the year before the local Stockholm recommendation, the Wise List, advised apixaban as the preferred NOAC.
