# Supplementary figures and images for: Factors associated with antithrombotic treatment decisions for stroke prevention in atrial fibrillation in the Stockholm region after the introduction of NOACs
Source: Eur J Clin Pharmacol. 2017 Jun 29;73(10):1315–22. doi: 10.1007/s00228-017-2289-0 (PMC5612279; doi:10.1007/s00228-017-2289-0)

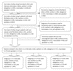

Supplement: Supplementary file 3 — Flow chart of patient selection (GIF 1 kb) [file 228_2017_2289_Fig2_ESM.gif]

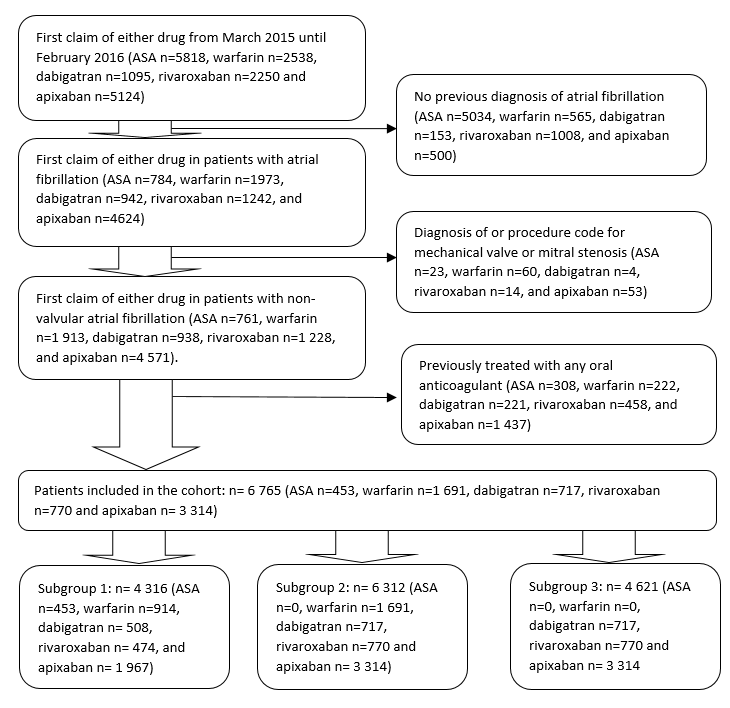

Supplement: Supplementary file 4 — High Resolution Image (TIFF 78 kb) [file 228_2017_2289_MOESM3_ESM.tif]

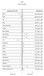

Supplement: Appendix Figure 2 — Adjusted Odds Ratios (aOR) of all comorbidities associated with treatment choises for (A) ASA compared to an oral anticoagulant, (B) warfarin compared to NOAC, (C) dabigatran compared to apixaban and rivaroxaban, (D) rivaroxaban compared to dabigatran and apixaban, and (E) apixaban compared to dabigatran and rivaroxaban. In this Figure the multivariate model for analyzing the effects of age on treatment decisions is shown. This model includes all complicating comorbidities as defined in Table 1. (GIF 941 bytes) [file 228_2017_2289_Fig3_ESM.gif]

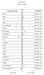

Supplement: Appendix Figure 2 — Adjusted Odds Ratios (aOR) of all comorbidities associated with treatment choises for (A) ASA compared to an oral anticoagulant, (B) warfarin compared to NOAC, (C) dabigatran compared to apixaban and rivaroxaban, (D) rivaroxaban compared to dabigatran and apixaban, and (E) apixaban compared to dabigatran and rivaroxaban. In this Figure the multivariate model for analyzing the effects of age on treatment decisions is shown. This model includes all complicating comorbidities as defined in Table 1. (GIF 941 bytes) [file 228_2017_2289_Fig4_ESM.gif]

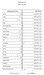

Supplement: Appendix Figure 2 — Adjusted Odds Ratios (aOR) of all comorbidities associated with treatment choises for (A) ASA compared to an oral anticoagulant, (B) warfarin compared to NOAC, (C) dabigatran compared to apixaban and rivaroxaban, (D) rivaroxaban compared to dabigatran and apixaban, and (E) apixaban compared to dabigatran and rivaroxaban. In this Figure the multivariate model for analyzing the effects of age on treatment decisions is shown. This model includes all complicating comorbidities as defined in Table 1. (GIF 941 bytes) [file 228_2017_2289_Fig5_ESM.gif]

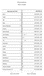

Supplement: Appendix Figure 2 — Adjusted Odds Ratios (aOR) of all comorbidities associated with treatment choises for (A) ASA compared to an oral anticoagulant, (B) warfarin compared to NOAC, (C) dabigatran compared to apixaban and rivaroxaban, (D) rivaroxaban compared to dabigatran and apixaban, and (E) apixaban compared to dabigatran and rivaroxaban. In this Figure the multivariate model for analyzing the effects of age on treatment decisions is shown. This model includes all complicating comorbidities as defined in Table 1. (GIF 941 bytes) [file 228_2017_2289_Fig6_ESM.gif]

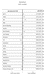

Supplement: Appendix Figure 2 — Adjusted Odds Ratios (aOR) of all comorbidities associated with treatment choises for (A) ASA compared to an oral anticoagulant, (B) warfarin compared to NOAC, (C) dabigatran compared to apixaban and rivaroxaban, (D) rivaroxaban compared to dabigatran and apixaban, and (E) apixaban compared to dabigatran and rivaroxaban. In this Figure the multivariate model for analyzing the effects of age on treatment decisions is shown. This model includes all complicating comorbidities as defined in Table 1. (GIF 941 bytes) [file 228_2017_2289_Fig7_ESM.gif]

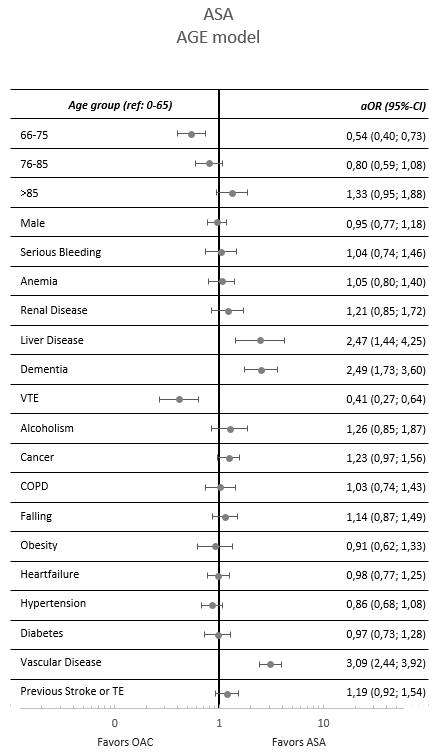

Supplement: Supplementary file 10 — High Resolution Image (TIFF 34 kb) [file 228_2017_2289_MOESM4_ESM.tif]

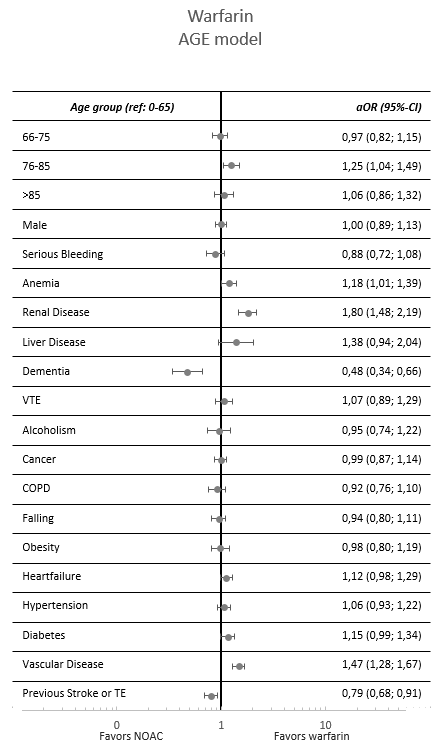

Supplement: Supplementary file 11 — High Resolution Image (TIFF 35 kb) [file 228_2017_2289_MOESM5_ESM.tif]

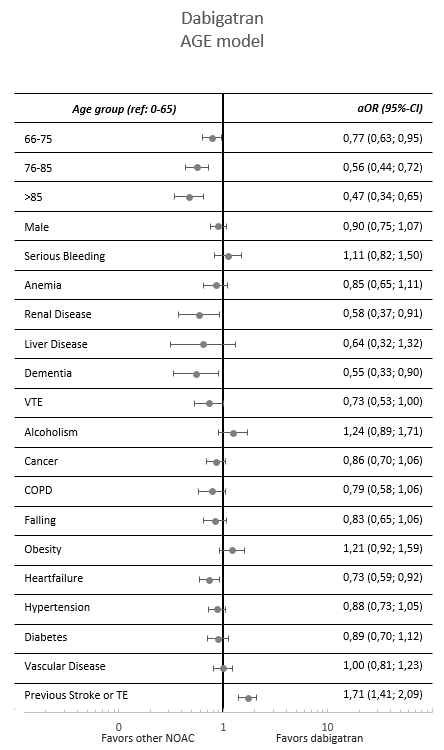

Supplement: Supplementary file 12 — High Resolution Image (TIFF 35 kb) [file 228_2017_2289_MOESM6_ESM.tif]

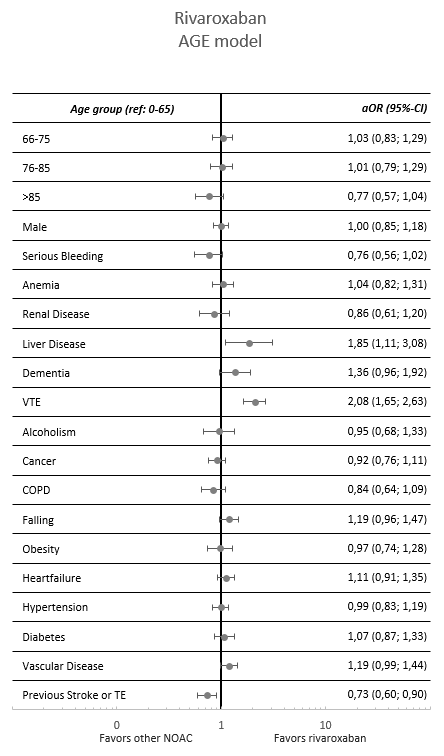

Supplement: Supplementary file 13 — High Resolution Image (TIFF 34 kb) [file 228_2017_2289_MOESM7_ESM.tif]

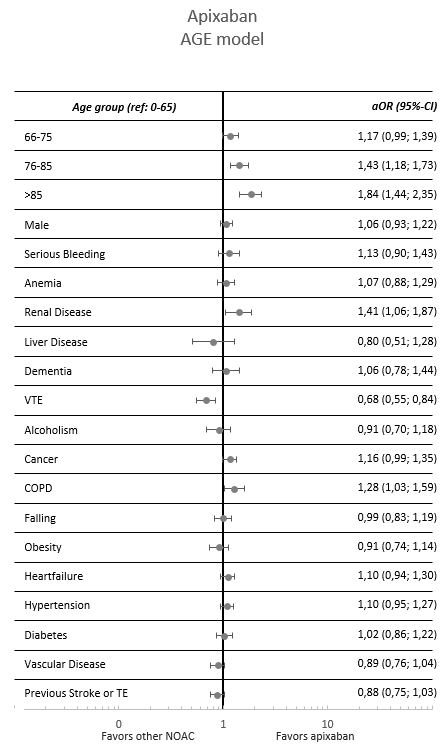

Supplement: Supplementary file 14 — High Resolution Image (TIFF 34 kb) [file 228_2017_2289_MOESM8_ESM.tif]
